# Contribution of adenosine A_2A_ receptor agonist and antagonist on ovarian ischemia/reperfusion injury in rats

**DOI:** 10.55730/1300-0144.6170

**Published:** 2025-12-31

**Authors:** Azad MAMMADOV, Abdullah Tuncay DEMİRYÜREK, Ahmet SARACALOĞLU, Cahit DEMİRKIRAN, İsmail Tuncer DEĞİM, Ömer ERONAT, Şeniz DEMİRYÜREK

**Affiliations:** 1 Department of Medical Pharmacology, Faculty of Medicine, Gaziantep University, Gaziantep, Turkiye; 2 Department of Pharmaceutical Technology, Faculty of Pharmacy, Biruni University, İstanbul, Turkiye; 3 Department of Medical Pathology, Faculty of Medicine, Gaziantep University, Gaziantep, Turkiye; 4 Department of Physiology, Faculty of Medicine, Gaziantep University, Gaziantep, Turkiye

**Keywords:** Adenosine receptor, ischemia/reperfusion, istradefylline, ovary, regadenoson

## Abstract

**Background/aim:**

To determine the effects of an A_2A_ receptor agonist regadenoson, an A_2A_ receptor antagonist istradefylline, and istradefylline nanosuspension on ovarian ischemia/reperfusion (I/R) injury in rats.

**Materials and methods:**

A total of 80 female rats were divided into 10 groups: sham, ovarian I/R, blank nanosuspension + ovarian I/R, regadenoson (3 μg/kg) + ovarian I/R, regadenoson (30 μg/kg) + ovarian I/R, istradefylline (0.3 mg/kg) + ovarian I/R, istradefylline (3 mg/kg) + ovarian I/R, istradefylline-loaded nanosuspension (3 mg/kg) + ovarian I/R, istradefylline (3 mg/kg) + regadenoson (30 μg/kg) + ovarian I/R, and istradefylline-loaded nanosuspension (3 mg/kg) + regadenoson (30 μg/kg) + ovarian I/R. ELISA, chemiluminescence, and spectrophotometric analyses were performed on blood and ovarian tissue samples. Histopathological changes were analyzed using hematoxylin and eosin staining, and a scoring system was applied to assess ovarian tissue damage.

**Results:**

Serum malondialdehyde levels were augmented in the I/R and blank nanosuspension + I/R groups. Although tissue superoxide dismutase levels were decreased with I/R, these levels were increased with regadenoson (3 μg/kg), istradefylline (0.3–3 mg/kg), and istradefylline nanosuspension (3 mg/kg). While there was augmentation in the serum and tissue 3-nitrotyrosine levels in the I/R group, a marked decrease was identified with regadenoson (30 μg/kg, p < 0.05). Native thiol levels were decreased in the I/R group, and this decrease was prevented by regadenoson. Serum disulfide levels were increased in the I/R group, and this increase was suppressed by regadenoson.

**Conclusion:**

These data showed that adenosine A_2A_ receptors may contribute to the ovarian I/R injury and regadenoson can produce protective effects.

## 1. Introduction

Ovarian torsion is an acute obstetric and gynecologic emergency in which ischemic changes occur due to complete or partial rotation of the ligaments that supply and support the ovarian tissue, leading to a reduction or cessation of ovarian blood flow [1,2]. Adnexal torsion can lead to ovarian hypoxia, ischemia, edema, congestion, and hemorrhage. These findings vary in accordance with the duration of torsion and the degree of ovarian ischemia [3]. Although the incidence of ovarian torsion remains poorly determined, data imply ovarian torsion accounts for approximately 2.7% of surgical gynecologic emergencies [4,5]. Ovarian torsion often presents with an acute onset of pelvic pain, nausea, and vomiting. However, the treatment and diagnosis are frequently delayed due to the nonspecificity of the clinical presentations [5]. Ovarian torsion can affect women at any age, from the intrauterine period to the postmenopausal phase. However, it is most commonly observed in women of reproductive age [5,6]. Factors predisposing them to ovarian torsion encompass the presence of ovarian cysts, the manifestation of ovarian hyperstimulation syndrome, and pregnancy. Ovarian torsion during pregnancy typically occurs more frequently in the first trimester, and a major risk factor is the induction of ovulation [6,7]. The main approach to treating ischemic ovarian tissue involves untwisting the ovaries and restoring blood flow to the damaged tissue, even in cases where the tissue appears necrotic [3]. Ovarian ischemia leads to the initiation of a series of events called reperfusion, with reoxygenation of the tissue. During reperfusion, elevated oxygen levels result in increased reactive oxygen species (ROS), proinflammatory cytokines, activation of neutrophils and platelets, nitric oxide (NO), and apoptosis that damage endothelial cells [8,9]. As a result, reperfusion-related oxidative damage, defined as ischemia/reperfusion (I/R) injury, occurs in the ovaries [8].

Adenosine is increased in cellular stress states such as hypoxia, ischemia, stress, seizures, pain, trauma, and inflammation. It contributes to the several various physiological and pathophysiological events in tissues [10,11]. Adenosine receptor agonists and antagonists have been linked to numerous significant physiological functions, including neuromodulation, immune regulation, vascular function, and metabolic regulation [10,12]. Cellular stress leads to an elevation in extracellular adenosine levels, resulting in several physiological effects including the modulation of vasodilation, cardiac rhythm, lipid metabolism, immunological activities, sleep patterns, angiogenesis, and antiinflammatory responses [12–14]. Currently, only one selective adenosine A_2A_ agonist, regadenoson, and one selective A_2A_ antagonist, istradefylline, have been approved by the Food and Drug Administration as a pharmacological agent for myocardial perfusion imaging and as add-on therapy to levodopa in patients with Parkinson’s disease, respectively [15]. However, there is no published research related to the effects of an adenosine receptor agonist or antagonist on ovarian I/R injury. Hence, the purpose of the current study was to assess the contribution of the adenosine A_2A_ receptor in ovarian I/R injury in a rat model. It was also aimed to show the effect of istradefylline nanosuspension on ovarian I/R injury in this model.

## 2. Materials and methods

### 2.1. Preparation of nanosuspension

In the preparation of nanosuspension formulations, 5 mL of ethanol and 650 mg of Eudragit RS 100 (Evonik Nutrition & Care GmbH, Essen, Germany) were dissolved in separate beakers at room temperature. Following the dissolution process, 0.5 mg/mL of istradefylline and 5 mL of dimethyl sulfoxide (DMSO) were added into one of the beakers. The other beaker was left as blank formulation. The contents of beakers 1 and 2 were slowly added dropwise to 100 mL of distilled water in an ice bath containing 0.02% Tween 80. While adding the drops, they were mixed at 1500 *g*, and the resulting nanoformulations were kept at a low temperature for a short period of time. The slow evaporation of the organic solvent led to the polymer precipitating, forming micro- and nanodroplets as well as matrix-type nanoparticles. The zeta potential of the prepared nanoparticles was measured using a Litesizer 500 (Anton Paar, Graz, Austria) device.

After the istradefylline-loaded nanosuspensions were diluted, they were dried and coated using gold-palladium (Au-Pd) for 150 s using an Emitech SC7620 Mini Sputter Coater/Glow Discharge System (Quorum Technologies LTD., Laughton, UK). Then, high-resolution images were taken of the surface of the sample with a scanning electron microscope (SEM) (GeminiSEM 300, Carl Zeiss Microscopy GmbH, Corp., Oberkochen, Germany) at the university. The pore size dimensions were measured using ImageJ (version 1.54d) software [16].

### 2.2. Animals

This research was performed in the Gaziantep University Experimental Animals Research Center following the guidelines of the Guide for the Care and Use of Laboratory Animals and approved by the Gaziantep University Local Ethics Committee for Animal Research, under decision number 2022/47. The animals used in the study comprised 80 female Wistar albino rats aged 6–8 weeks and weighing 160–240 g. They were kept at a controlled room temperature of 22–25 °C, exposed to a 12-h light cycle, and had ad libitum access to food and water.

### 2.3. Study groups

The 80 rats were randomly divided into the following 10 experimental groups, and all treatments were administered intraperitoneally:

Sham group (sham, n = 8): saline and DMSO were administered as vehicles for regadenoson [17] and istradefylline [18], respectively.
Ovarian I/R group (I/R, n = 8): saline and DMSO were administered. To study the I/R injury, ovarian ischemia induced for 2 h, followed by a 2-h reperfusion period was applied, as previously described [19].
Blank nanosuspension + ovarian I/R group (Blank nano + I/R, n = 8): blank nanosuspension was administered 1 h before I/R.
Regadenoson (3 μg/kg) + ovarian I/R group (Rega 3 + I/R, n = 8): regadenoson (3 μg/kg) was administered 1 h before I/R.
Regadenoson (30 μg/kg) + ovarian I/R group (Rega 30 + I/R, n = 8): regadenoson (30 μg/kg) was administered 1 h before I/R.
Istradefylline (0.3 mg/kg) + ovarian I/R group (Ist 0.3 + I/R, n = 8): istradefylline (0.3 mg/kg) was administered 1 h prior to I/R.
Istradefylline (3 mg/kg) + ovarian I/R group (Ist 3 + I/R, n = 8): istradefylline (3 mg/kg) was administered 1 h prior to I/R.
Istradefylline-loaded nanosuspension group (3 mg/kg) + ovarian I/R (Nano Ist 3 + I/R, n = 8): istradefylline-loaded nanosuspension (3 mg/kg) was administered 1 h prior to I/R.
Istradefylline (3 mg/kg) + regadenoson group (30 μg/kg) + ovarian I/R (Ist 3 + Rega 30 + I/R, n = 8): istradefylline (3 mg/kg) was administered 10 min prior to regadenoson (30 μg/kg), and ovarian I/R was induced 1 h after that.
Istradefylline-loaded nanosuspension (3 mg/kg) + regadenoson (30 μg/kg) + ovarian I/R group (Nano Ist 3 + Rega 30 + I/R, n = 8): istradefylline-loaded nanosuspension (3 mg/kg) was administered 10 min prior to regadenoson (30 μg/kg), and ovarian I/R was induced 1 h after that.

Regadenoson (3–30 μg/kg, MedChemExpress LLC, Monmouth Junction, NJ, USA) and istradefylline doses (0.3–3 mg/kg, ABCR GmbH, Karlsruhe, Germany) were selected from previously published studies [17,20] and were administered intraperitoneally 1 h before ischemia. In the combination groups, istradefylline was administered 10 min prior to regadenoson. Upon completion of the experiment, intracardiac blood samples were promptly collected under anesthesia (administered intraperitoneally with 10 mg/kg of xylazine hydrochloride and 100 mg/kg of ketamine hydrochloride), and then bilateral oophorectomy was performed. Following this, biochemical and histopathological analyses were conducted.

### 2.4. Surgical procedure

After sterilizing the abdomens with iodine, a 2-cm lower midline vertical incision was made. A complete ovarian ischemia model was created by tying off the left ovarian arteries with a two-sided atraumatic silk suture in a top knot style for 2 h. The abdominal incision was then sutured closed using 3/0 silk sutures. Afterward, the silk sutures were removed to induce reperfusion for another 2 h. The incised abdomen skin was closed with a 3/0 silk suture. Following the experiment duration, samples of blood were quickly collected from within the heart by intracardiac puncture under anesthesia, and then the ovaries were carefully removed. All procedures were conducted during the daytime under ketamine/xylazine anesthesia in a sterile laboratory condition.

### 2.5. Biochemical analyses

The blood samples underwent centrifugation at 5000 *g* for 10 min at 4 °C. Next, the serum specimens were aliquoted, transferred to Eppendorf tubes, and stored at a temperature of −80 °C until analysis. Ovarian tissues frozen at −150 °C were thawed gradually and washed with cold phosphate buffered saline (PBS) to eliminate blood cells. Then, tissues were transferred into 2-mL cryovial tubes on ice. The protease inhibitor was added to the tissue following the kit procedure with a ratio of 9 mL of PBS per 1 g of tissue before storing it in a freezer at −20 °C. Once frozen, the tissue was homogenized for 3 min in a TissueLyser LT (Qiagen GmbH, Hilden, Germany), kept cold in a refrigerator, and then subjected to homogenization and vortexing. The tissue was homogenized using an ultrasonic homogenizer (Sonopuls, Bandelin, Germany) for 25–30 s, vortexed, and subsequently subjected to centrifugation at 5000 g for a duration of 10 min to separate the supernatants. The obtained tissue lysates were stored at −80 °C until analysis.

### 2.6. Enzyme-linked immunosorbent assay (ELISA)

Serum malondialdehyde (MDA) levels were measured using a commercially supplied ELISA kit (Cat. No. E0156Ra, BT LAB, Bioassay Technology Laboratory, Shanghai, China). Tissue superoxide dismutase (SOD) levels were determined using an ELISA kit (Cat. No. ER0332, rat SOD1, Cu-Zn Superoxide Dismutase, FineTest, Wuhan Fine Biotech Co., Ltd., Wuhan, Hubei, China) following the instructions provided by the manufacturer. The levels of 3-nitrotyrosine in serum and tissue were measured using an ELISA kit (Cat. No. E0019Ra, rat 3-nitrotyrosine, 3-NT, BT LAB, Bioassay Technology Laboratory). All absorbances were measured spectrophotometrically using the Epoch Microplate Spectrophotometer (BioTek Instruments, Winooski, VT, USA) at 450 nm.

### 2.7. NO analysis

Serum and tissue lysates were analyzed for nitrite and nitrate levels using a chemiluminescence technique, as described previously [21]. The NO/ozone chemiluminescence technique measured the gaseous form of NO. Serum samples were analyzed using a Sievers Model 280i NOA chemiluminescence system (Sievers Instruments, Boulder, CO, USA).

### 2.8. Thiol/disulfide detection

Serum and tissue lysate thiol/disulfide levels were measured spectrophotometrically using commercially available kits for analysis (Rel Assay Diagnostics, Mega Tip Ltd., Gaziantep, Türkiye) as published previously [22]. Measurements included both serum native thiols (reduced thiols, –SH) and total thiols (including both –SH and dynamic disulfide bonds, –S–S–). The dynamic disulfide content was calculated as half of the difference between the total thiols and the native thiols.

### 2.9. Histopathological analysis

After fixing the ovaries in 10% formaldehyde solution, each ovarian tissue sample was fixed in paraffin. Hematoxylin and eosin (H&E) were used to stain paraffin-embedded ovarian tissue samples. For measurements, the slides were examined and photographed using a Nikon Eclipse E600 (Nikon Instruments Inc., Melville, NY, USA) microscope. Histological slides were evaluated for vascular congestion, interstitial edema, polymorphonuclear (PMN) infiltration, and hemorrhage. The scoring, as described by Eser et al. [18], was applied on a scale from 1 to 4, where 1 signifies no presence; 2 indicates mild; 3 denotes moderate; and 4 represents severe. Grade 1 is characterized by mild vascular congestion, no PMN, no hemorrhage, and mild edema. Grade 2 comprises moderate vascular congestion, no PMN, no hemorrhage, and moderate edema. Grade 3 involves severe vascular congestion, severe edema, mild PMN infiltration, and mild hemorrhage. Grade 4 indicates severe vascular congestion, severe edema, severe PMN infiltration, and severe hemorrhage.

### 2.10. Statistical analysis

The data were presented as the mean ± standard error of the mean, standard deviation (SD), or percentage. Statistical analyses were performed using GraphPad Instat (version 3.05, GraphPad Software Inc., San Diego, CA, USA) and IBM SPSS Statistics for Windows 20.0 (IBM Corp., Armonk, NY, USA). The Kolmogorov–Smirnov normality test was employed to assess the normal distribution of the variables, revealing that all variables adhered to a normal distribution (p > 0.05). The unpaired Student’s t test was utilized to analyze the means of two groups, and the chi-squared test was utilized to test the differences in percentages. The means of more than two groups were compared using analysis of variance, followed by either the post hoc Student–Newman–Keuls or Dunnett test. The Kruskal–Wallis test, followed by the post hoc Dunn’s test, was applied to compare the histopathological scores among the different groups. The Pearson correlation test was conducted for correlation analysis. Significance was accepted as p < 0.05 for all statistical tests.

## 3. Results

### 3.1. MDA and SOD levels

**Figure 1:**
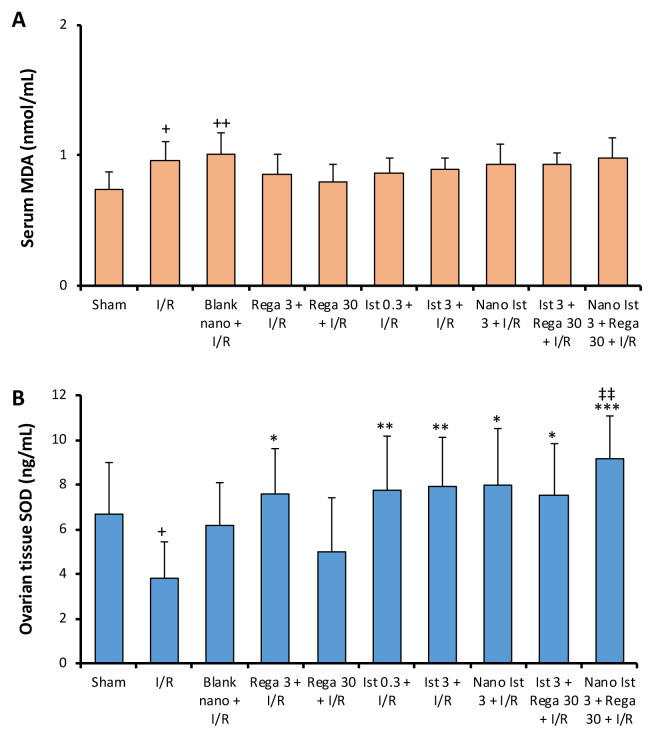
Serum MDA and tissue Cu-Zn SOD levels of the study groups (n = 8 for each). Results are given as the mean ± standard error of the mean. + p < 0.05, ++ p < 0.01 compared to the sham group, * p < 0.05, ** p < 0.01, *** p < 0.01 compared with the I/R group, ‡‡ p < 0.01 compared with the regadenoson (30 μg/kg) + I/R group.

Serum MDA levels were markedly elevated in both the I/R and Blank nano + I/R groups compared to the Sham group (Figure 1). Tissue Cu-Zn superoxide dismutase (SOD) levels were markedly decreased in the I/R group compared to the Sham group. Tissue Cu-Zn SOD levels measured in the Rega 3 + I/R, Nano Ist 3 + I/R, and Ist 3 + Rega 30 + I/R groups were higher than the I/R group. Tissue Cu-Zn SOD levels in the Ist 0.3 + I/R and Ist 3 + I/R groups demonstrated higher tissue Cu-Zn SOD levels than the I/R group. In addition, tissue Cu-Zn SOD levels were higher in the Nano Ist 3 + Rega 30 + I/R group compared to the Rega 30 + I/R group.

### 3.2. NO and 3-NT levels

**Figure 2:**
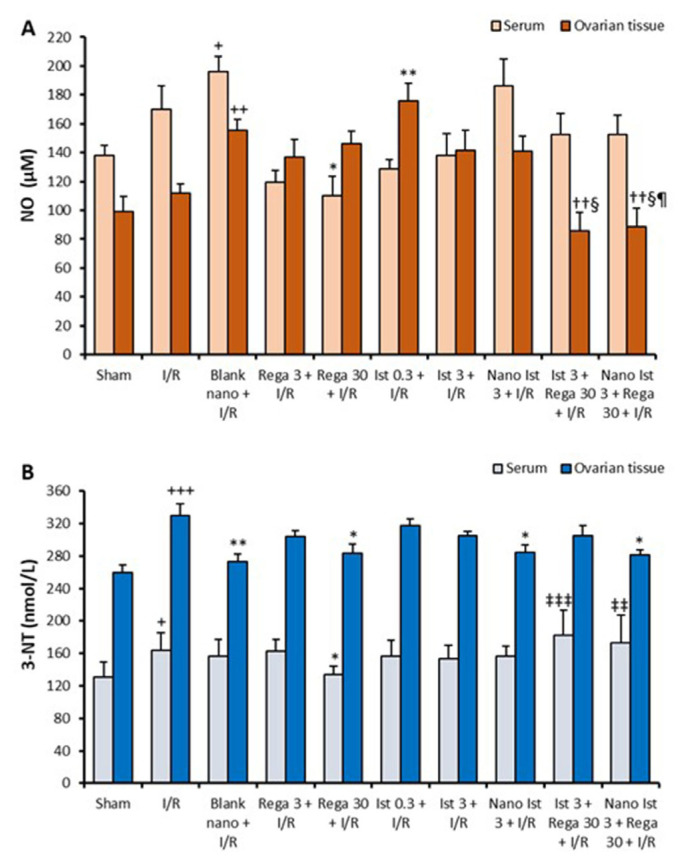
Serum and tissue levels of NO (A) and 3-NT (B) for the study groups (n = 8 for each). Results are shown as the mean ± standard error of the mean. + p < 0.05, ++ p < 0.01, +++ p < 0.001 compared to the sham group, * p < 0.05, ** p < 0.01 compared to the I/R group, ‡‡ p < 0.01, ‡‡‡ p < 0.001 compared to the regadenoson (30 μg/kg) + I/R group, †† p < 0.01 compared to the blank nanoformulation + I/R group, § p < 0.05 compared to the istradefylline 3 mg/kg + I/R group, ¶ p < 0.05 compared to the nano istradefylline 3 mg/kg + I/R group.

Serum and tissue NO levels of the study groups are given in Figure 2. The serum NO level of the Blank nano + I/R group was significantly elevated compared to the Sham group. Conversely, the serum NO levels in the Rega 30 + I/R group were markedly decreased compared to those in the I/R group. Furthermore, tissue NO levels in the Blank nano + I/R group were substantially higher than in the Sham group. Moreover, tissue NO levels in the Ist 0.3 + I/R group were markedly increased compared to the I/R group. The combination treatment of regadenoson with istradefylline decreased the tissue NO levels, suggesting that a combination of treatments was more successful in decreasing tissue, but not serum, levels of NO.

The serum and tissue levels of 3-NT for the groups are presented in Figure 2. The serum 3-NT level was increased in the I/R group compared to the Sham group. The serum 3-NT level of the Rega 30 + I/R group was significantly decreased compared to the I/R group. Additionally, the serum 3-NT levels of both the Ist 3 + Rega 30 + I/R and Nano Ist 3 + Rega 30 + I/R groups were higher than the Rega 30 + I/R group. Additionally, the tissue 3-NT levels of the I/R group were increased compared to the Sham group. Treatment with regadenoson 30 μg/kg decreased the serum and tissue 3-NT levels (Figure 2).

### 3.3. Thiol-disulfide levels

**Figure 3:**
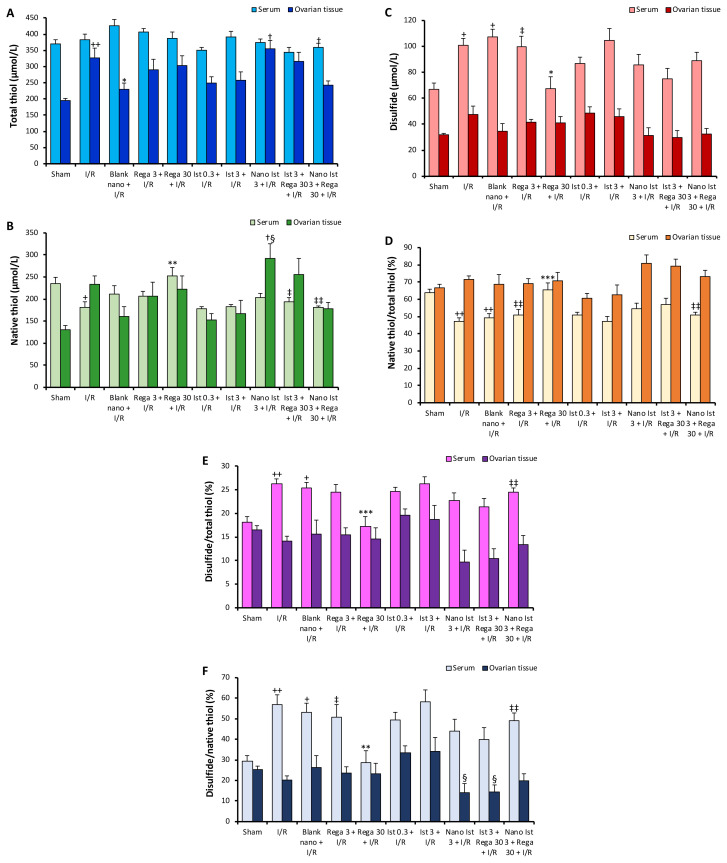
(A–F) Effects of adenosine A_2A_ receptor agonist and antagonist on dynamic thiol/disulfide homeostasis in ovarian I/R injury (n = 8, for each). (A) total thiol levels, (B) native thiol levels, (C) disulfide levels, (D) native thiol/total thiol ratio, (E) disulfide/total thiol ratio, (F) disulfide/native thiol ratio. Results are given as the mean ± standard error of the mean. + p < 0.05, ++ p < 0.01 compared to the sham group, * p < 0.05, ** p < 0.01, *** p < 0.001 compared to the I/R group, ‡ p < 0.05, ‡‡ p < 0.01 compared to the regadenoson (30 μg/kg) + I/R group. † p < 0.05 compared to the blank nanoformulation + I/R group, § p < 0.05 compared to the istradefylline 3 mg/kg + I/R group.

The thiol-disulfide homeostasis in both serum and tissue across the different groups is shown in Figure 3. The serum total thiol levels were significantly diminished in the Nano Ist 3 + Rega 30 + I/R group compared to the Blank nano + I/R group. The tissue total thiol level was significantly increased in the I/R group compared to the Sham group. In the Blank nano + I/R group, the tissue total thiol ratio was lower than in the (I/R group. Furthermore, the tissue total thiol ratio was significantly increase in the Ist 3 + Rega 30 + I/R group compared to the Blank nano + I/R group (Figure 3A).

The I/R group showed a substantial decrease in serum native thiol levels compared to the Sham group. The serum levels of native thiol in the Rega 30 + I/R group were significantly increased compared to the I/R group. The serum native thiol levels in the Rega 30 + I/R group were significantly increased compared to the Ist 3 + Rega 30 + I/R group. Moreover, the Rega 30 + I/R group exhibited markedly higher serum native thiol levels than the Nano Ist 3 + Rega 30 + I/R group. Additionally, tissue native thiol levels were higher in the Nano Ist 3 + I/R group compared to the Blank nano + I/R and Ist 3 + I/R groups (Figure 3B).

Serum disulfide levels in the I/R and Blank nano + I/R groups were significantly increased compared to the Sham group. Conversely, the Rega 30 + I/R group exhibited significantly lower serum disulfide levels than the I/R group. Additionally, serum disulfide levels in the Rega 3 + I/R group were significantly higher than those in the Rega 30 + I/R group. However, no significant differences were detected in the tissue disulfide levels between the groups (Figure 3C).

Serum native thiol/total thiol ratios were lower in the I/R and Blank nano + I/R groups compared to the Sham group. This ratio was markedly higher in the Rega 30 + I/R group compared to the I/R group. The native thiol/total thiol ratio decreased in the I/R group, and this decrease was prevented with the high-dose regadenoson. Istradefylline pretreatment abolished the effects of regadenoson. However, no marked changes were noted in the tissue native thiol/total thiol ratios between the groups (Figure 3D).

In the Rega 30 + I/R group, the serum disulfide/total thiol ratio was markedly lower than in the I/R group. In addition, the serum disulfide/total thiol ratio was higher in the Nano Ist 3 + Rega 30 + I/R group compared to the Rega 30 + I/R group. However, there was no statistical significance in the tissue disulfide/total thiol ratio between the groups (Figure 3E).

Regadenoson (30 μg/kg) pretreatment decreased the serum disulfide/native thiol ratio. The levels of serum disulfide/total thiol and disulfide/native thiol ratios were markedly increased in the I/R group. However, these increases were suppressed when the high-dose regadenoson was administered (Figure 3F).

### 3.4. Correlation analysis

Statistically significant positive and negative correlations between the parameters measured in ovarian I/R experiments in rats are presented in Tables 1 and 2, respectively. In the Rega 3 + I/R group, a negative correlation was found between the serum native thiol levels and serum disulfide levels (Table 2). Moreover, a positive correlation was found between the tissue total thiol levels and tissue native thiol levels (Table 1). In the Rega 30 + I/R group, a positive correlation was noted between the tissue total thiol levels and tissue native thiol levels (Table 1). A positive correlation was revealed between the serum total thiol levels and serum disulfide levels in the Ist 0.3 + I/R group (Table 1). In this group, a negative correlation was found between the serum native thiol levels and tissue NO levels (Table 2).

### 3.5. SEM analysis, particle size, and zeta potential measurements

**Figure 4:**
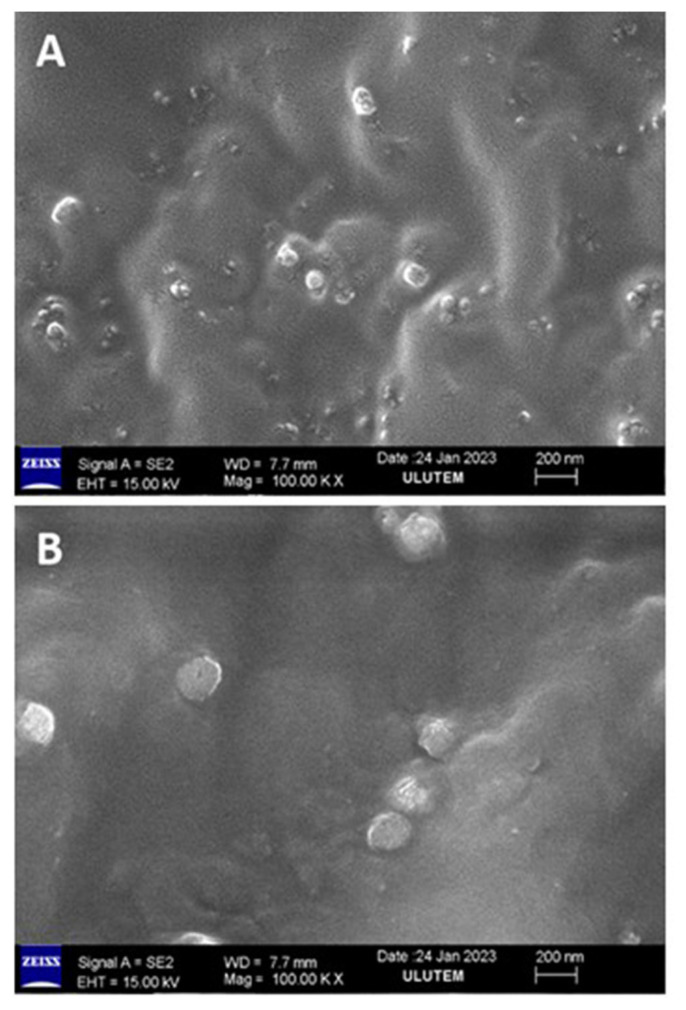
SEM micrographs of the nanosuspensions. (A) Blank and (B) istradefylline-loaded.

The size of particles and the morphology of the formulated blank and drug-loaded nanosuspensions were characterized using a SEM. The SEM images show the spherical morphology of the prepared blank and istradefylline-loaded nanosuspensions (Figure 4). The average particle size in the blank nanosuspension was 111.07 ± 21.40 nm, and the average particle size in the istradefylline-loaded nanosuspension was 224.52 ± 52.61 nm. All zeta potential measurements were repeated 10 times, and the zeta potential for istradefylline nanosuspension was 18.0 ± 0.025 mV.

### 3.6. Histopathological examination

**Figure 5:**
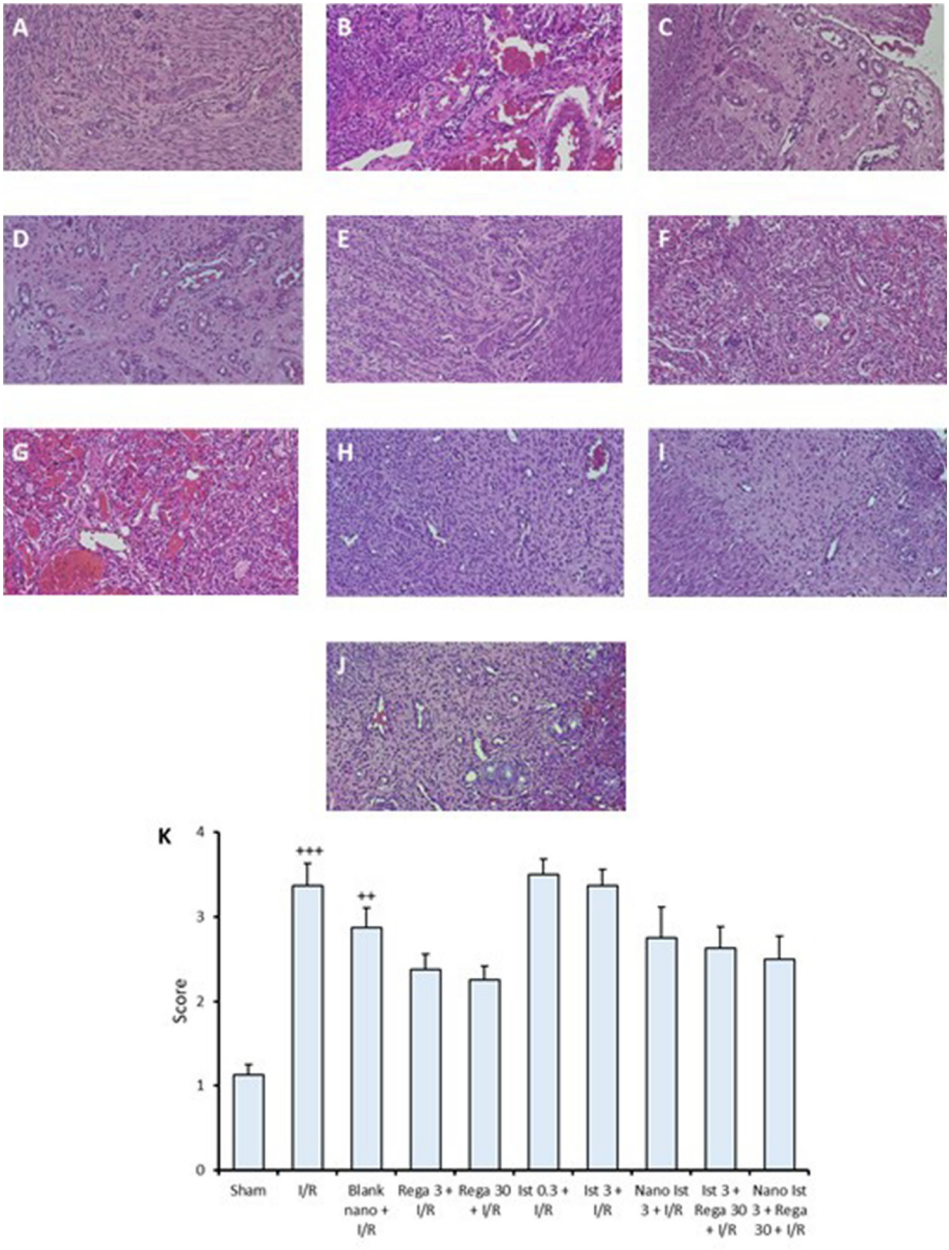
(A–J) Representative histopathological images of the ovarian tissues. (A) Sham group, (B) I/R group, (C) Blank nanosuspension + I/R group, (D) regadenoson 3 μg/kg + I/R group, (E) regadenoson 30 μg/kg + I/R group, (F) istradefylline 0.3 mg/kg + I/R group, (G) istradefylline 3 mg/kg + I/R group, (H) istradefylline-loaded nanosuspension 3 mg/kg + I/R group, (I) regadenoson (30 μg/kg) + istradefylline (3 mg/kg) + I/R group, (J) regadenoson (30 μg/kg) + istradefylline-loaded nanosuspension (3 mg/kg) + I/R group, (K) total histopathological score of the study groups. ++ p < 0.01, +++ p < 0.001 when compared to the sham group, All H&E staining was ×200

Representative histopathological images of ovarian tissues and total scores are presented in Figure 5. In the sham group, histopathological examination revealed generally preserved ovarian tissue with mild PMN infiltration, and no signs of interstitial edema, vascular congestion, or hemorrhage were determined. In contrast, the I/R group exhibited severe interstitial edema, PMN infiltration, hemorrhage, and vascular congestion. The Blank nano + I/R group showed moderate PMN infiltration and mild interstitial edema, without signs of vascular congestion or hemorrhage. The Rega 3 + I/R group exhibited mild PMN infiltration and severe interstitial edema. The Rega 30 + I/R group displayed mild PMN infiltration and moderate interstitial edema, with no signs of vascular congestion or hemorrhage. The Ist 0.3 + I/R group exhibited vascular congestion, severe interstitial edema, mild hemorrhage, and severe PMN infiltration. The Ist 3 + I/R group showed severe PMN infiltration, moderate vascular congestion, severe interstitial edema, and mild hemorrhage. The Nano Ist 3 + I/R group exhibited hemorrhage, severe interstitial edema, mild PMN infiltration, and moderate vascular congestion. In the Ist 3 + Rega 30 + I/R group, severe vascular congestion, interstitial edema, moderate hemorrhage, and mild PMN infiltration were observed. The Nano Ist 3 + Rega 30 + I/R group showed moderate vascular congestion, moderate interstitial edema, and mild PMN infiltration, with no significant hemorrhage findings (Figure 5). The histopathologic scores of the I/R and Blank nano + I/R group were higher than the Sham group. No significant change was recorded between the other groups (Figure 5).

## 4. Discussion

This study is the first to show that adenosine A_2A_ receptor agonist regadenoson produced a protective effect in a rat model of ovarian I/R injury. Adenosine A_2A_ receptor antagonist istradefylline was able to inhibit the effects of regadenoson in these experiments. Istradefylline nanosuspension formulation was prepared for the first time with this study. Nanosuspension delivery aims to target the tissue, lower toxicity, and rapid access to the therapeutic dose at the site of action [23]. Recent reports have identified that the use of the nanosuspension form offers numerous advantages, including targeted delivery, controlled release, high stability, and relatively low toxicity [24,25]. The results herein demonstrated similar activities as istradefylline except, that nanoformulation produced higher native thiol levels and markedly suppressed the disulfide/native thiol ratio.

Although the gold standard for management of ovarian torsion is surgical detorsion of the adnexa, several interventions have been described. It has been demonstrated that hyperbaric oxygen treatment or hypothermia (4 °C for 30 min) can inhibit the production of oxidative stress in the ovaries subjected to torsion/detorsion injury in rats [26,27]. Additionally, exogenous administration of some hormones, such as melatonin, leptin, or erythropoietin has been shown to attenuate the severity of ovarian I/R injury [28–30]. The present study investigated the role of adenosine receptor modulation on ovarian I/R injury in rats.

Regadenoson and istradefylline were used at doses of 3–30 μg/kg and 0.3–3 mg/kg, respectively. Regadenoson is administered at a dose of 8–10 μg/kg in clinical studies [31,32]. In clinical studies, it is recommended to start istradefylline at a dose of 20 mg once daily and increase to 40 mg once daily as tolerated [33]. In one study, istradefylline was used at a dose of 20 mg/day, escalating to 40 mg/day for patients weighing an average of 52 kg [34], which amounted to doses of approximately 0.4–0.8 mg/kg. An 80-mg dose was also used in another study [35]. Therefore, the doses used in human studies are within the range of the doses used in the current study.

Herein, it was found that adenosine A_2A_ receptor stimulation generates preventive effects against ovarian I/R injury in rats. These results support the data showing that activation of A_2A_ receptors before reperfusion saves cellular survival, improves ventricular contraction disorders, and alleviates myocardial I/R injury in rats [36]. Another study indicated that a selective A_2A_ receptor agonist significantly attenuates systemic I/R injury in a porcine model of circulatory arrest and extracorporeal cardiopulmonary resuscitation [37]. This study is also in line with the results of the present study. It has been shown that adenosine maintains microvascular blood flow, inhibits neutrophils and the resulting inflammatory cascade, stabilizes cell membranes, reduces the formation of free radicals, provides antiinflammatory effects, provides calcium homeostasis, and has an effect on many reperfusion injury mechanisms [10,38]. Adenosine A_2A_ receptor shows a heightened sensitivity to hypoxia, as it is transcriptionally stimulated by hypoxia-induced factor 1α (HIF-1α) [39].

Herein, serum MDA levels were investigated to evaluate I/R injury by lipid peroxidation products. The serum MDA levels of the group that underwent I/R after administration of blank nanosuspension and the group that underwent only I/R were higher than the sham group, and both increases were significant. The formation of MDA due to lipid peroxidation has been described in ovarian I/R studies [40,41]. In the present study, the augmentation in serum MDA levels recorded in the I/R group suggests that lipid peroxidation and thus cell damage may be the cause of the injury.

Oxygen-derived free radicals formed during I/R are released in tissues and can be scavenged by SOD. SOD is an important antioxidant enzyme that catalyzes the conversion of superoxide radicals to H_2_O_2_. In the present research, SOD levels were noted to be low in the I/R but significantly elevated in the other groups. These findings are in agreement with the observation that SOD level is depressed during I/R [40,41]. When the combined treatment using nanosuspension was compared with regadenoson alone, the tissue SOD level was higher in the combined nano-treatment group.

Nitrosative stress is accepted to be closely linked with oxidative stress [42]. Several studies have shown that 3-NT formation is a specific biomarker of nitrosative stress [43]. In the present study, serum and tissue 3-NT levels were significantly decreased with the high dose of regadenoson, suggesting that this drug can inhibit the nitrosative stress.

Our data showed that increased serum NO levels during I/R can contribute to the nitrosative stress, which was inhibited with high-dosage regadenoson. The main NOS isoforms found in rat ovaries are inducible NO synthase (iNOS) and endothelial NOS (eNOS) [44]. Previous studies on ovarian I/R injury in rats found a downregulation in eNOS expression and an upregulation in iNOS expression in the I/R group [45,46]. Increased intracellular levels of NO may trigger toxic processes that result in cell death, mostly by producing ROS and generating more damaging substances like peroxynitrite [43]. Reperfusion injury is known to be mediated by ROS formed via lipid peroxidation. The thiol/disulfide homeostasis is crucial for maintaining the cellular redox status and plays a role in various pathologies [47]. Increased serum disulfide levels, serum disulfide/total thiol ratio, and serum disulfide/native thiol ratio in the I/R group were markedly decreased by high-dose regadenoson. These data suggest that regadenoson has an antioxidant effect.

Since there was a positive correlation between tissue thiol levels, the protective effects of regadenoson appeared to be related to increased thiol levels. Positive correlations of istradefylline treatment with serum thiol and NO levels suggest that istradefylline was not able to suppress oxidative or nitrosative stress in ovarian I/R injury. On the other hand, negative correlations observed with istradefylline imply that increased 3-NT and NO levels may induce thiol depletion and produce imbalance in thiol-disulfide homeostasis.

There were several limitations of this study. The main limitation of the study was that the differences between groups were largely not statistically significant in the histopathology analysis. Although the histological scores appeared to be modified with the regadenoson or istradefylline treatments, these differences did not reach a statistically significant level. Another limitation was related to serum MDA levels. Neither regadenoson nor istradefylline was able to induce changes in serum MDA levels. These findings could have been related to the small sample size of the groups.

In conclusion, this study is the first to investigate the effects of adenosine A_2A_ receptor agonist regadenoson and adenosine A_2A_ receptor antagonist istradefylline, along with istradefylline-nanoformulation, on ovarian I/R injury. The oxidative and nitrosative stress-reducing effects of the adenosine A_2A_ receptor agonist regadenoson were observed. The results demonstrated that regadenoson treatment showed a protective effect against ovarian damage in rat I/R injury. The use of an adenosine A_2A_ receptor agonist could be an alternative or new therapeutic approach for the management of ovarian torsion.

## Figures and Tables

**Table 1:** Significant positive correlations between the measured parameters of the groups.

Parameters	Correlation coefficient (r)	Coefficient of determination (r^2^)	p
*In regadenoson 3 μg/kg + I/R group:*			
Tissue total thiol ↔ Tissue native thiol	0.9946	0.9893	<0.0001
*In regadenoson 30 μg/kg* ↔ *I/R group:*			
Tissue total thiol ↔ Tissue native thiol	0.9504	0.9912	0.0003
*In istradefylline 0.3 mg/kg + I/R group:*			
Serum total thiol ↔ Serum disulfide	0.8770	0.7691	0.0042
*In istradefylline 3 mg/kg + I/R group:*			
Serum total thiol ↔ Serum disulfide	0.9740	0.9487	<0.0001
Tissue total thiol ↔ Tissue native thiol	0.9120	0.8317	0.0016
*In nano-istradefylline 3 mg/kg + I/R group:*			
Serum total thiol ↔ Serum disulfide	0.7842	0.6149	0.0212
Serum disulfide ↔ Serum NO	0.8982	0.8068	0.0024
Serum disulfide ↔ Tissue SOD	0.9462	0.8953	0.0013
Tissue total thiol ↔ Tissue native thiol	0.9418	0.8870	0.0005
*In regadenoson (30 μg/kg) + istradefylline (3 mg/kg) + I/R group:*			
Serum total thiol ↔ Serum disulfide	0.8217	0.6751	0.0124
Tissue total thiol ↔ Tissue native thiol	0.9677	0.9365	<0.0001
Tissue native thiol ↔ Tissue NO	0.7123	0.5074	0.0474
*In regadenoson (30 μg/kg) + Nano-istradefylline 3 mg/kg + I/R group:*			
Serum total thiol ↔ Serum MDA	0.8974	0.8054	0.0025
Serum total thiol ↔ Serum 3-NT	0.7587	0.5756	0.0291
Serum total thiol ↔ Serum disulfide	0.9643	0.9298	0.0001
Serum disulfide ↔ Serum MDA	0.8661	0.7501	0.0054
Tissue total thiol ↔ Tissue native thiol	0.7885	0.6217	0.0201

NO: nitric oxide, 3-NT: 3-nitrotyrosine, MDA: malondialdehyde, SOD: superoxide dismutase.

**Table 2:** Significant negative correlations between the measured parameters of the groups.

Parameters	Correlation coefficient (r)	Coefficient of determination (r^2^)	p
*In regadenoson 3 μg/kg + I/R group:*			
Serum native thiol ↔ Serum disulfide	−0.7194	0.5176	0.0442
*In istradefylline 0.3 mg/kg + I/R group:*			
Serum native thiol ↔ Tissue NO	−0.7288	0.5311	0.0403
*In istradefylline 3 mg/kg + I/R group:*			
Serum total thiol ↔ Tissue 3-NT	−0.7929	0.6287	0.0189
Serum disulfide ↔ Tissue total thiol	−0.7751	0.6008	0.0239
Serum disulfide ↔ Tissue 3-NT	−0.7133	0.5089	0.0470
*In nano-istradefylline 3 mg/kg + I/R group:*			
Serum native thiol ↔ Tissue SOD	−0.8374	0.7012	0.0187
Serum native thiol ↔ Serum 3-NT	−0.7985	0.6376	0.0175
*In regadenoson (30 μg/kg) + istradefylline (3 mg/kg) + I/R group:*			
Tissue disulfide ↔ Tissue NO	−0.9470	0.8968	0.0004
Serum NO ↔ Tissue SOD	−0.8596	0.7389	0.0062
*In regadenoson (30 μg/kg) + Nano-istradefylline 3 mg/kg + I/R group:*			
Tissue native thiol ↔ Tissue SOD	−0.7853	0.6167	0.0209
